# Transfer of Xenomitochondria Containing the Entire Mouse Mitochondrial Genome into a Genetically Modified Yeast Expressing Mitochondrial Transcription Factor A

**DOI:** 10.4014/jmb.2004.04033

**Published:** 2020-07-05

**Authors:** Young Geol Yoon

**Affiliations:** Department of Biomedical Science, Jungwon University, Goesan-gun, Chungbuk 28024, Republic of Korea

**Keywords:** Xenomitochondria, mtDNA, mitochondrial transcription factor A, Tfam, spheroplast, N- ethylmaleimide

## Abstract

Recently, it was reported that entire mammalian mtDNA genomes could be transplanted into the mitochondrial networks of yeast, where they were accurately and stably maintained without rearrangement as intact genomes. Here, it was found that engineered mtDNA genomes could be readily transferred to and steadily maintained in the mitochondria of genetically modified yeast expressing the mouse mitochondrial transcription factor A (Tfam), one of the mitochondrial nucleoid proteins. The transferred mtDNA genomes were stably retained in the Tfam-expressing yeast cells for many generations. These results indicated that the engineered mouse mtDNA genomes introduced in yeast mitochondria could be relocated into the mitochondria of other cells and that the transferred genomes could be maintained within a mitochondrial environment that is highly amenable to mimicry of the biological conditions in mammalian mitochondria.

## Introduction

Mitochondria are intracellular organelles present in the cytoplasm of eukaryotic cells. As a unique feature, these organelles have their own genome, mitochondrial DNA (mtDNA). In most mammalian cells, the circular mtDNA genomes are approximately 16.5 kb in size and encode 13 polypeptide components of the ATP-producing pathway, as well as the tRNAs and rRNAs required for mitochondrial gene expression [1]. Despite their relatively small size, mtDNA genomes in animal cells are essential for sustaining normal cellular function, and mutations in these genomes can cause a wide array of incurable diseases and other complex conditions, such as cancer, diabetes and neurodegeneration, mediated through mitochondrial dysfunction [2, 3]. Although the critical importance of mtDNA genomes to normal cellular function and overall human health is now well understood, the lack of a practical method to genetically modify mtDNA in animal cells prevents us from investigating various aspects of mtDNA and from modeling mitochondrial diseases caused by pathogenic mutations [4].

Recently, yeast cells that contained engineered animal mtDNA genomes within their mitochondrial networks were described, and the approach that was used to generate these cells was detailed [5]. Mouse mtDNA genomes that were engineered and replicated as plasmid clones in a bacterial host could be transplanted into the mitochondrial network of yeast cells, in which they were stably maintained and faithfully replicated as complete circular genomes [5]. Here, engineered mouse mtDNA genomes in the mitochondrial environment have been shown to be freely transferred to and maintained in genetically modified yeast mitochondria expressing the mouse mitochondrial transcription factor A (Tfam) protein instead of the yeast mitochondrial transcription factor Abf2p [6, 7]. Although the transferred mtDNA genomes were stably maintained in the strain expressing Abf2p, the mtDNA genomes in the strain expressing the mouse Tfam were relatively less stably maintained, which indicated that the mouse Tfam could partially support the replication of the mouse mtDNA genomes in yeast mitochondria. The results shown here demonstrate that the xenomitochondria carrying the engineered animal mtDNA genomes can be relocated from one cell to another in which crucial mitochondrial proteins are modified to be expressed without difficulty and that yeast mitochondria are a highly versatile host system for maintaining and analyzing mammalian mtDNA genomes within a mitochondrial environment in which the biological circumstances can be easily converted to those found in mammalian mitochondria.

## Materials and Methods

### Strains and Media

The yeast strain MCC109 ρ^0^ (*MATα, ade2-101, ura3-52, kar1-1,* ρ^0^) was obtained from Dr. Fox [8]. The 3482-16-1ρ^0^ (*MATa, ura3-52, leu2-3,112, trp1-289, his3-*Δ*1, met2, Cyh^R^,* ρ^0^) strain was obtained from Dr. Livingston (University of Minnesota) [9]. MCC109 ρ^+^ cells were generated by mating the MCC109 ρ^0^ cells with the AH109 (*MATa*, *trp1-901*, *leu2-3*, ρ^+^) strain and by screening haploid MCC109 cells [10]. The *E. coli* strain DH5α λ_*att*_::*pirwt* was used to maintain γ-*ori*-containing plasmids [11]. Total mouse cellular DNA was isolated from mouse STO embryonic fibroblasts (CRL-1503, ATCC). Preparation of complete medium containing glucose (YPD; 1% yeast extract, 2% peptone and 2% glucose), complete medium containing the nonfermentable carbon sources ethanol and glycerol (YPEG; 1% yeast extract, 2% peptone, 3% ethanol and 3% glycerol), synthetic minimal medium containing glucose (SD), and synthetic minimal medium containing the nonfermentable carbon sources ethanol and glycerol (SEG) and standard genetic manipulations of yeast were performed as previously described [5].

### Mitochondrial Transformation and DNA Analysis

Yeast mitochondrial transformation was carried out according to a published method using a biolistic transformation apparatus, PDS-1000/He (Bio-Rad) [8]. To screen mitochondrial transformants, a PCR assay was carried out using mouse mtDNA-specific primers [5, 12]. Total yeast DNA was fractionated in a 1% agarose gel and analyzed by Southern blotting using the complete mouse mtDNA as a probe.

### Construction of Yeast with a Mouse *Tfam* Insertion

The mouse *Tfam* gene was amplified by PCR from the mouse Tfam IMAGE clone 1890394 (Invitrogen, USA). To insert the *Tfam* gene into the yeast *ABF2* locus, sequences corresponding to the *ABF2* leader presequences followed by the coding portion of the mouse Tfam protein were included [10]. After obtaining the *Tfam-URA3* product by recombinant PCR, the product was directly transformed into the 3482-16-1ρ^0^ strain to replace the yeast *ABF2* with the mouse *Tfam* gene [10]. Deletion of *ABF2* was confirmed by PCR using the following primers: ABF2 internal (5’-GATAAATGGCAATCCTTGGATC-3’) and ABF2assay3’ (5’-GGTGAGGACGAGTTATGGTG-3’) [10]. The *Tfam* insertion was confirmed by PCR using total yeast DNA as a template with the Tfamforward and Tfamreverse primers or Tfamforward and ABF2assay3’ primers [10].

### Inactivation of Spheroplasts with Chemicals

The antifungal compound *N*-ethylmaleimide (EMI) was selected to inactivate yeast spheroplasts without altering their genetic composition or lysing them [13]. After lyticase treatment, the yeast spheroplasts were treated with EMI (50 μg/ml in 0.7 M KCl) for 1 h at RT and washed three times with 0.7 M KCl. The EMI-treated spheroplasts were then used for cell fusion with chemically untreated yeast spheroplasts. The reactivated cells obtained via spheroplast fusion were selected on minimal medium depending on the fusion partners.

### Spheroplast Formation and Yeast Cell Fusion

The method described by Kucsera *et al*. [13] was used with minor modifications. Yeast cells of the fusion partners were cultivated in YPD liquid medium, collected by centrifugation and pretreated in 2 volumes of reducing solution (0.02 M EDTA, 0.02 M Tris-HCl (pH 8.0), 0.1 M β-mercaptoethanol, 0.7 M KCl) for 15 min at 35°C to eliminate disulfide bonds on the cell wall. The yeast cell wall was then digested with lyticase (1 mg/ml) in 0.7 M KCl for 30 min at 35°C, and the resulting spheroplasts were washed by repeated centrifugation in 0.7 M KCl. For fusion, 10^8^ spheroplasts from each partner were mixed and treated with 2 ml of polyethylene glycol (PEG) solution (20% PEG 6000, 0.1 M CaCl_2_) for 30 min at RT. After removal of the PEG solution by centrifugation, the spheroplasts were diluted in 0.3 M CaCl_2_ and then embedded in 2% top agar containing 0.4 M CaCl_2_ as an osmotic stabilizer at 45°C. The mixture was overlaid onto presolidified selective minimal medium depending on the fusion partners.

## Results

### Insertion of the Mouse *Tfam* Gene at the Yeast *ABF2* Locus

**Fig. 1:**
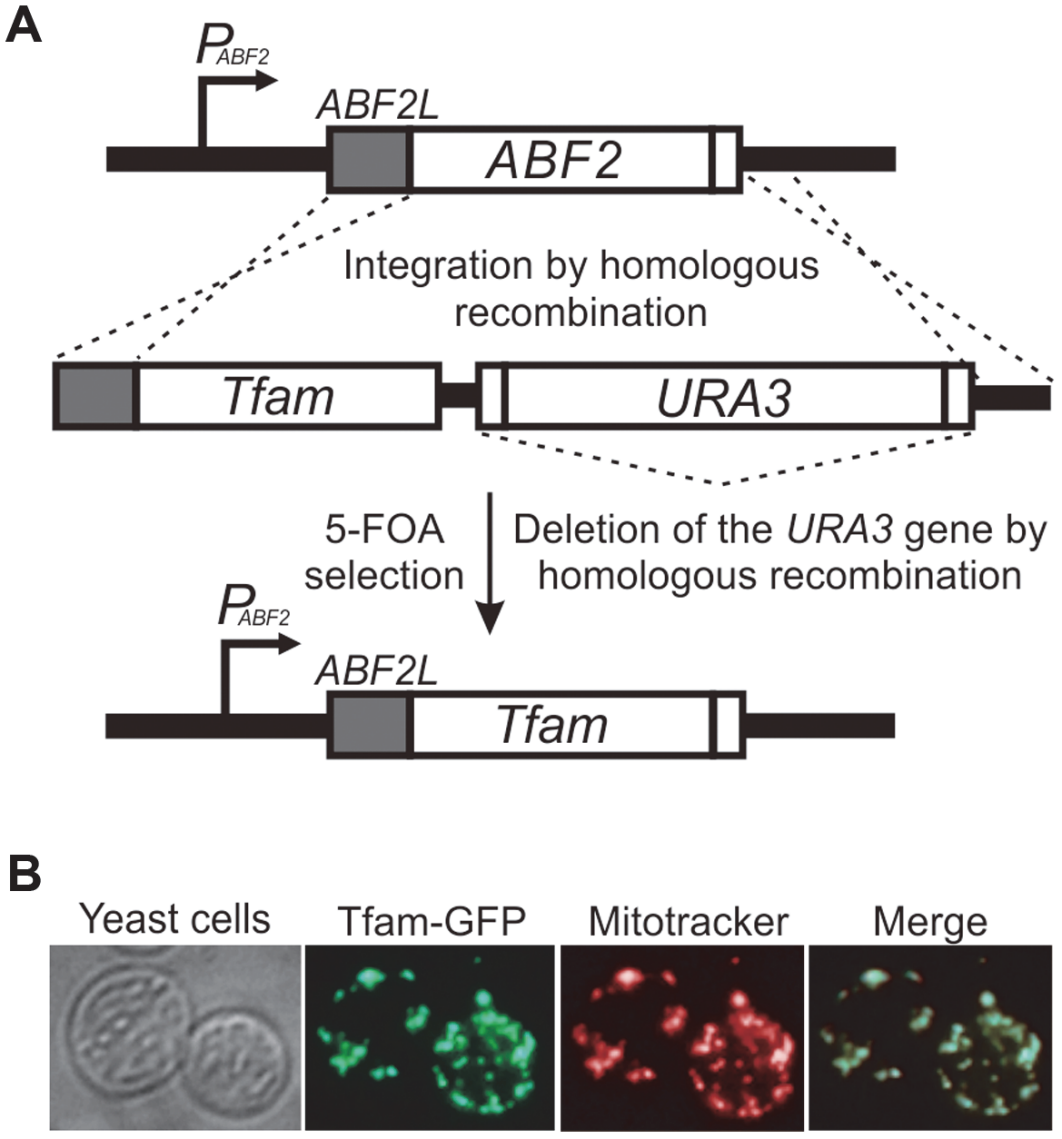
Construction of a yeast strain carrying the mouse *Tfam* gene at the yeast *ABF2* locus. (**A**) Schematic diagram of the mouse *Tfam* gene insertion at the *ABF2* site. The *Tfam* gene was integrated after the yeast *ABF2* mitochondrial leader sequence by homologous recombination. After correct integration was verified, the *URA3* selection marker gene was eliminated by 5-FOA-mediated homologous recombination. (**B**) Targeting of mouse Tfam protein to yeast mitochondria. The GFP-fused Tfam proteins were expressed in wild-type yeast cells and localized near the periphery of these cells with a punctate fluorescence pattern that exactly matched the MitoTracker-labeled mitochondria.

In this experiment, the yeast strain 3482-16-1 was genetically modified to express mouse mitochondrial transcription factor A (Tfam). The reasons for choosing this strain were that the strain contained four available selectable markers (*ura3, leu2, trp1* and *his3*) and that mtDNA-less ρ^0^ cells were available. Thus, this strain was advantageous for introducing additional genes related to mtDNA maintenance and expression using additional selectable markers in subsequent studies as well as for use as recipient ρ^0^ cells for transfer experiments. To introduce mouse *Tfam* into the yeast genome, the yeast *ABF2* gene was replaced with the mouse *Tfam* gene [10]. The mouse *Tfam* and the *URA3* selection marker were amplified by PCR separately, and recombinant PCR was performed to generate the *Tfam*-*URA3* fusion construct. Fifty and 52 homologous nucleotides at the 5’-end and 3’-UTR region of the yeast *ABF2* gene, respectively, were included in the 5’- and 3’-ends of the *Tfam*-*URA3* fusion construct, which could be used for insertion of the fusion product into the *ABF2* locus by homologous recombination (Fig. 1) [10]. The *Tfam-URA3* construct was transformed into yeast 3482-16-1 ρ^0^ cells, which have no mtDNA in their mitochondria, and Ura^+^ transformants were selected on uracil-deficient synthetic minimal medium containing glucose (SD/Ura^-^) (Fig. 1A). Insertion of the *Tfam-URA3* construct was confirmed by PCR and sequencing. The *Tfam-URA3*-containing Ura^+^ transformants were then cultured in YPD medium for 2 days and were spread on 5-fluoroorotic acid (FOA)-containing synthetic complete medium to remove the *URA3* selection marker by homologous recombination [14]. Since a sequence containing 36 homologous nucleotides (5’-CCTCTCAACTAGACCGCGGTACTCTCACAATGTTTT-3’) flanked by the *URA3* gene was added, the marker gene could be deleted by homologous recombination during FOA selection; therefore, a *Tfam-*inserted yeast strain, without *ABF2* and *URA3*, could be isolated [10].

To verify the targeting of mouse Tfam into yeast mitochondria, recombinant PCR was performed to generate a construct expressing an Abf2 leader sequence (ABF2L)-Tfam-GFP fusion protein (Fig. 1B). When this PCR construct was transformed into yeast 3482-16-1 ρ^0^ cells, the Tfam-GFP proteins were clearly expressed in these cells, exhibiting a punctate fluorescence pattern located near the periphery of the cells, which was similar to the typical morphological fluorescence pattern seen when expressing Abf2p-GFP [15, 16]. The punctate fluorescence pattern closely matched the pattern obtained by staining with a MitoTracker, which indicates that the mouse Tfam protein could be efficiently and correctly targeted to the mitochondria in yeast 3482-16-1 ρ^0^ cells.

### Functional Complementation of Yeast Abf2p with Mouse Tfam

**Fig. 2:**
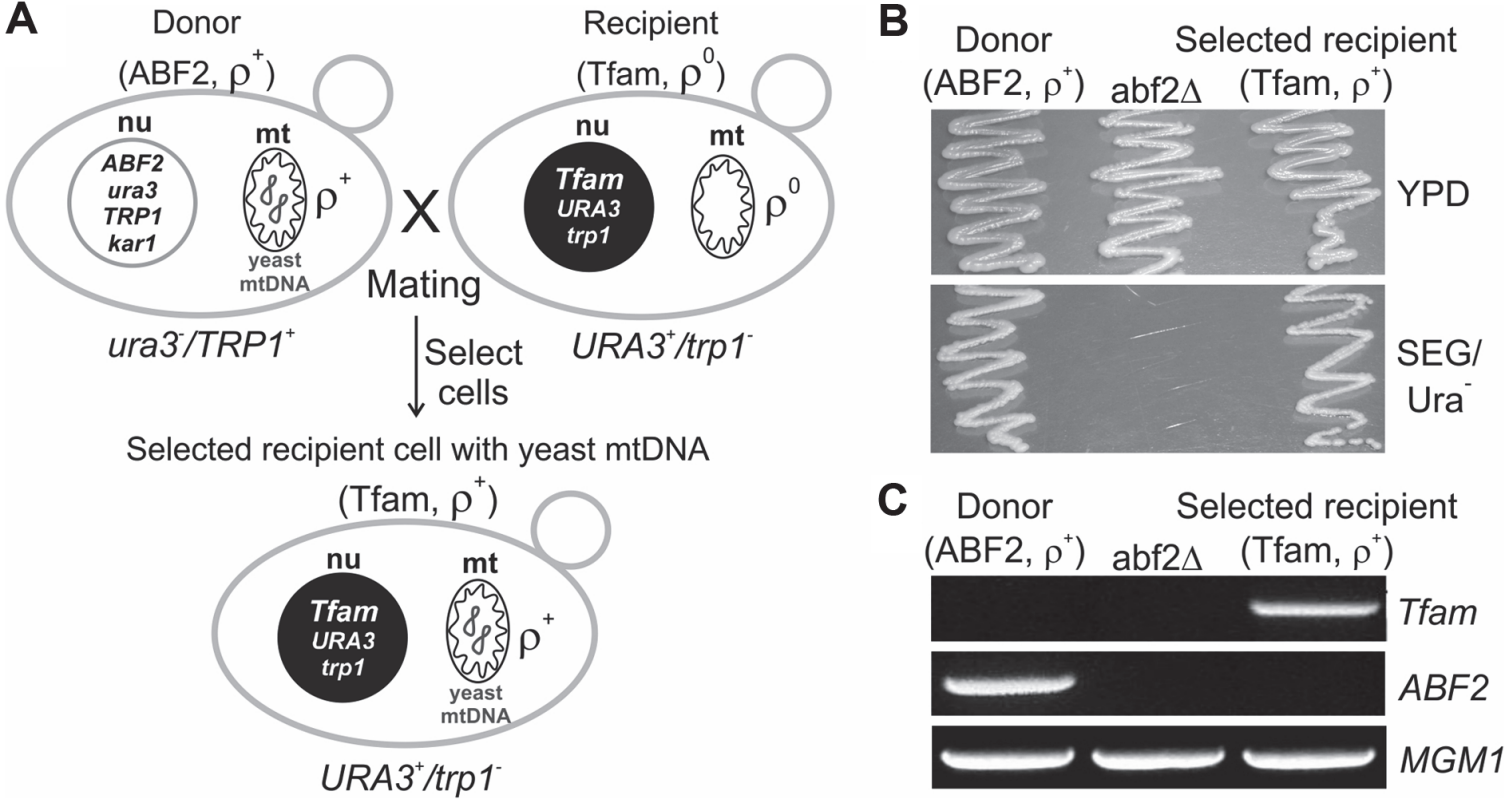
Assay for yeast mtDNA maintenance activity of the mouse Tfam protein in yeast mitochondria. (**A**) Construction of a yeast cell containing the mouse *Tfam* gene and full yeast mtDNA. The karyogamy-defective mutation (*kar1- 1*) and selection markers (*URA3* and *TRP1*) were used to screen yeast cells (Tfam, ρ^+^) that maintained active mitochondrial respiratory function after a genetic cross between the wild-type mtDNA donor (ABF2, ρ^+^) and Tfam ρ^0^ recipient cells. (**B**) Yeast cell growth on the two different growth media. The ABF2 wild-type, *abf2* deletion mutant (abf2Δ) and Tfam strains were cultured in glucose medium (YPD) and ethanol medium (SEG/Ura-), respectively. The abf2Δ strain could not grow on ethanol medium because the strain lacked active mitochondrial function to use ethanol as the carbon source for growth due to the loss of mtDNA. In contrast, the Tfam strain was able to grow on ethanol medium because the strain recovered the active mitochondrial function due to the mtDNA maintenance activity of the Tfam protein. (**C**) PCR analysis. Insertion of the *Tfam* gene and deletion of the *ABF2* gene were verified by PCR. As a control, a PCR assay for the yeast *MGM1* gene was performed.

It has been reported that the mouse *Tfam* genes introduced into the *ABF2* locus of the yeast genome and the corresponding mouse protein Tfam could functionally replace yeast Abf2p and support mtDNA maintenance and mitochondrial biogenesis in yeast [10]. In this work, the compensation of Abf2p function with mouse Tfam was verified using the yeast 3482-16-1 ρ^0^ strain to determine whether mouse Tfam can functionally replace yeast ABF2 activity (Fig. 2). The yeast mtDNA-maintaining ρ^+^ yeast strain was generated with the mouse *Tfam* gene insertion at the yeast *ABF2* gene locus as shown in the previous work [10]. The genetic cross (mating) between the wild-type strain and the *Tfam* gene-containing 3482-16-1 ρ^0^ strain (Tfam strain) was performed to produce haploid yeast cells, not diploids, maintaining the ρ^+^ yeast mtDNA in the presence of the mouse Tfam protein (Fig. 2A). As a wild- type strain, MCC109 ρ^+^ (*ura3*^-^/*TRP1*^+^), which cannot grow on uracil-deficient medium, was used as the yeast mtDNA donor. The Tfam strain (*URA3*^+^/*trp1*^-^) was used as a recipient strain that has no active mitochondrial function due to the absence of mtDNA and thus cannot grow on the medium with the nonfermentable carbon sources ethanol and glycerol.

The Tfam strain (*MATa*) was mated with yeast MCC109 ρ^+^ (*MATα*) wild-type cells. The mating mixtures were streaked on a synthetic uracil-deficient minimal medium containing the nonfermentable carbon sources ethanol and glycerol (SEG/Ura^-^). On this selection medium, both types of cells, haploid (3482-16-1 *abf2*Δ::*Tfam*, ρ^+^) and diploid (3482-16-1 *abf2*Δ::*Tfam* + MCC109 *ABF2*, ρ^+^), can be obtained because the strain MCC109 carries a karyogamy-defective mutation (*kar1-1*), which allows efficient mitochondrial fusion but greatly reduces nuclear fusion [17]. To identify whether the mated cells were haploids or diploids, each of the yeast colonies grown on SEG/Ura^-^ medium was tested by restreaking on a synthetic tryptophan-deficient minimal medium containing ethanol and glycerol (SEG/Trp^-^). Because the donor strain (MCC109 ρ^+^) can grow on SEG/Trp^-^ medium (Fig. 2A), any cells that can grow on both SEG/Ura^-^ and SEG/Trp^-^ media after mating are diploids that are able to survive only by the production of a diploid state. Therefore, haploid Tfam cells carrying intact yeast mtDNA (ρ^+^), which can grow only on SEG/Ura^-^ medium but not on SEG/Trp^-^ medium, could be selectively isolated by this screening method. As shown in Fig. 2B, recipient Tfam ρ^+^ cells were able to propagate on SEG/Ura^-^ medium, which indicated that the cells could retain active mitochondrial function owing to the yeast mtDNA maintenance activity of the mouse Tfam protein. In addition, the *Tfam* gene insertion and the *ABF2* gene deletion in the Tfam strain were confirmed by PCR analysis (Fig. 2C).

To determine how strongly the mouse Tfam protein supports the maintenance of yeast mtDNA in yeast mitochondria, MCC109 ρ^+^ Tfam cells were generated; these cells have an *ade* mutation, producing red colonies when they have active mitochondrial function but white colonies when they lose mitochondrial function [18]. MCC109 ρ^+^ wild-type and MCC109 ρ^+^ Tfam cells were first grown on synthetic minimal medium containing the nonfermentable carbon sources ethanol and glycerol (SEG) and were switched to cultivation on complete medium containing glucose (YPD) (Figs. S1A and S1B). The red colonies shown by the MCC109 ρ^+^ Tfam cells (Fig. S1B) rapidly disappeared and turned white (Fig. S1D), while the MCC109 wild-type ρ^+^ cells maintained the red colonies on both growth media (Figs. S1A and S1C). When these white colonies (Fig. S1D) along with red colonies (Fig. S1B) were streaked back on the SEG medium, the white colonies failed to grow, while the red colonies survived on this medium (data not shown). These results reflect the results of a previous report that showed that the full human *TFAM* and the mouse *Tfam* genes can functionally replace *ABF2* in yeast but are not as efficient as the yeast homolog at maintaining the full ρ^+^ yeast mtDNA genome [10, 19]. Mitochondrial function in the Tfam strain was lost at a higher rate than that in the yeast Abf2p wild-type strains when the cells were subjected to growth on nonselective complete medium containing glucose (Figs. S1C and S1D).

### Transfer of Xenomitochondria Containing Complete Mouse mtDNA Genomes into the Tfam Strain

**Fig. 3:**
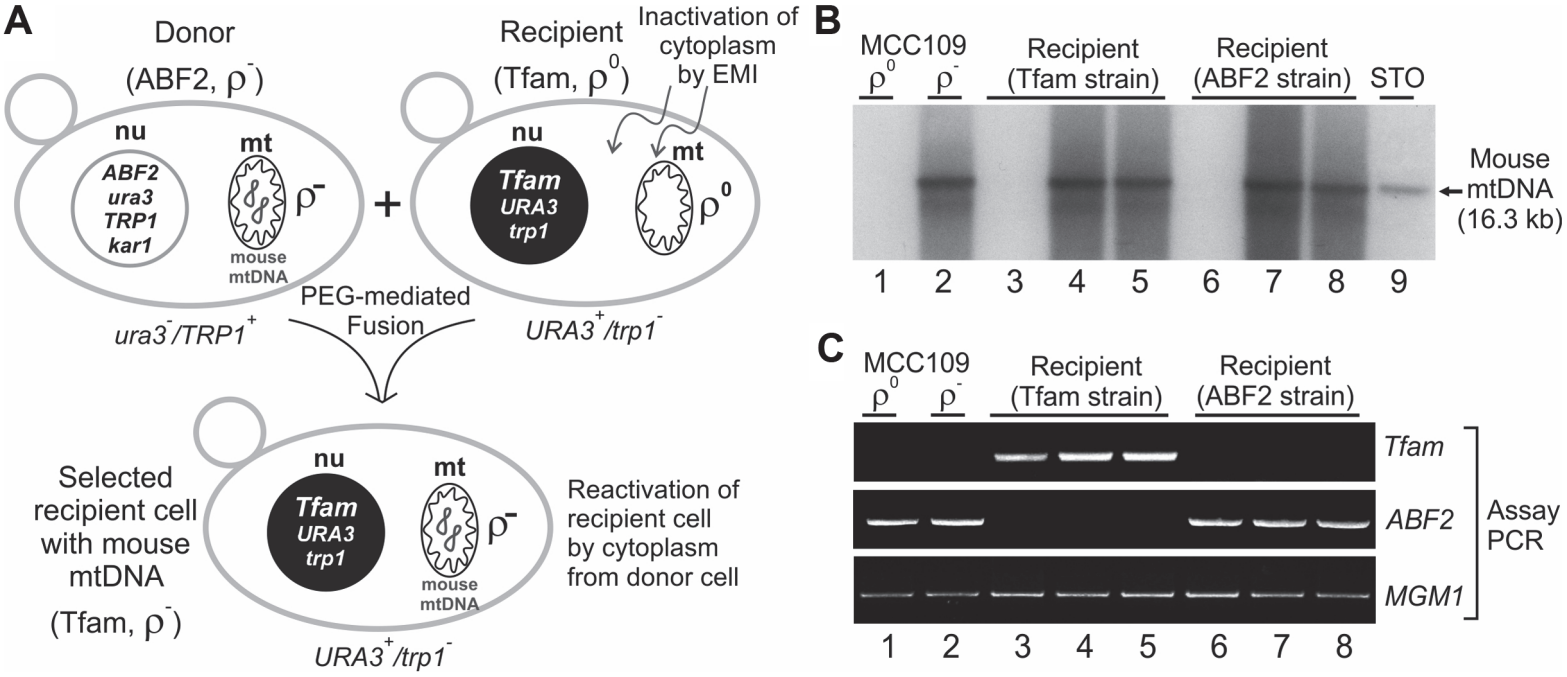
Transfer of xenomitochondria containing complete mouse mtDNA by chemical inactivation and reactivation of spheroplasts. (**A**) Construction of a yeast cell containing the mouse *Tfam* gene and complete mouse mtDNA. The karyogamy-defective *kar1-1* mutation and selection markers (*URA3* and *TRP1*) were used to isolate yeast cells (Tfam, ρ^-^) that maintained mouse mtDNA after PEG-mediated spheroplast fusion between the mouse mtDNA donor (ABF2, ρ^-^) and Tfam ρ^0^ recipient cells. (**B**) Southern blot analysis. Total DNA was isolated from yeast MCC109 ρ^0^ (no mtDNA control) (lane 1), from the donor MCC109ρ^-^ strain that contained the mouse mtDNA (lane 2), from recipient Tfam ρ^0^ (lane 3) and reactivated recipient Tfam ρ^-^ (lanes 4 and 5) cells, from recipient ABF2 ρ^0^ (lane 6) and reactivated recipient ABF2 ρ^-^ (lanes 7 and 8) cells, and from mouse STO cells (lane 9), digested with XhoI, and analyzed by electrophoresis and Southern blotting using the radiolabeled mouse mtDNA probe. The restriction fragments from the mtDNA maintained in yeast mitochondria matched those from the mtDNA from mouse cells (arrow). (**C**) PCR analysis. Insertion of the *Tfam* gene and deletion of the *ABF2* gene in the recipient Tfam strain were verified by PCR. As a control, a PCR assay for the yeast *MGM1* gene was performed. Lane 1, MCC109 ρ^0^; lane 2, MCC109 ρ^-^; lane 3, recipient Tfam ρ^0^; lanes 4 and 5, reactivated recipient Tfam ρ^-^; lane 6, recipient ABF2 ρ^0^; lanes 7 and 8, reactivated recipient ABF2 ρ^-^.

To test whether the genetically modified yeast strain was able to maintain the entire mouse mtDNA genome in its mitochondrial network, xenomitochondria containing the mouse mtDNA genomes were relocated to the 3482-16-1 Tfam strain along with the 3482-16-1 wild-type ABF2 strain (Fig. 3). EMI (50 μg/ml) was used to inactivate the yeast spheroplasts that were generated by the lyticase treatment. The EMI-treated recipient spheroplasts were fused with donor spheroplasts in which xenomitochondria containing the mouse mtDNA genomes were incorporated by the PEG-mediated fusion method to avoid altering their genetic composition or lysing them (Fig. 3) [13].

The donor spheroplasts (*ura3*^-^/*TRP1*^+^) could not grow on a synthetic uracil-deficient selection medium, but the recipient spheroplasts (*URA3*^+^/*trp1*^-^) could survive on this medium. Thus, chemically inactivated recipient spheroplasts could be reactivated only by the functional cytoplasm from the donor spheroplasts through spheroplast fusion (Fig. 3A). Using this spheroplast fusion method, both haploid and diploid cells could be obtained because the donor cells carry the *kar1-1* mutation [17]. By streaking the fused cells on synthetic uracil- and tryptophan-deficient selection media, the diploid cells that could grow on both media could be removed, and the haploid Tfam strain that could grow on only synthetic uracil-deficient selection medium could be isolated. Fig. 3B shows that the complete mouse mtDNA genomes were stably maintained in the Tfam recipient strain (lanes 4 and 5) as they were in the MCC109 wild-type donor cells (lane 2) and ABF2 recipient strain (lanes 7 and 8). By PCR analyses, the *Tfam* gene insertion (Fig. 3C, Tfam panel, lanes 3-5) and the yeast *Abf2* gene deletion (Fig. 3C, ABF2 panel, lanes 3-5) were verified. It should be noted that the ABF2 recipient strain contained only the ABF2 wild-type gene (Fig. 3C, ABF2 panel, lanes 6-8). Introduction of xenomitochondria containing the mouse mtDNA genomes directly into the abf2Δ mutant was attempted by using the EMI fusion method, but unfortunately, colonies that retained the mouse mtDNA in this abf2 deletion background were not obtained. Based on these results, it was concluded that the yeast mitochondria containing the mouse mtDNA genomes could be transferred to the other yeast cells (Tfam strain) and that the transferred mammalian mtDNA genomes could be faithfully maintained with the help of the mitochondrial genome-supporting activity of the mouse Tfam protein.

### Stability of Mouse mtDNA Genomes in the Tfam Strain

**Fig. 4:**
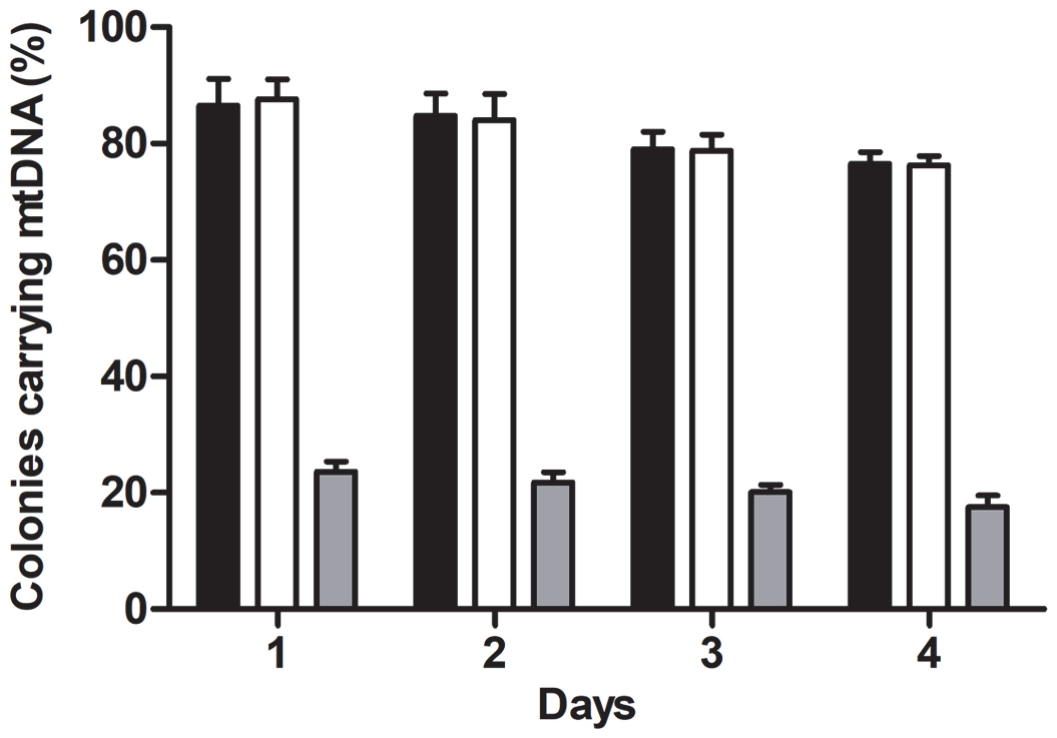
Mouse mtDNA stability in yeast strains. Yeast cells carrying the entire mouse mtDNA were sequentially cultured in glucose media by reinoculating the previous cultures into fresh media for four days. Mouse mtDNA stability was expressed as the percentage of colonies maintaining mouse mtDNA. More than 30 colonies that had formed on the YPD plates were directly assayed by PCR to identify the existence of the mtDNA. Black bars, wild-type ABF2 donor strains (MCC109 ρ^-^ with mouse mtDNA); white bars, wild-type ABF2 recipient strains (3482-16-1 ρ^-^ with mouse mtDNA); gray bars, recipient Tfam strains (3482-16-1 *abf2*Δ::*Tfam* ρ^-^ with mouse mtDNA).

Whether the transferred mouse mtDNA genomes are stably maintained in the Tfam strain as well as in the wild- type ABF2 recipient strain was tested (Fig. 4). Yeast colonies from each of the strains containing the mouse mtDNA genomes were inoculated into YPD media. These cultures were grown overnight in a shaking incubator at 30°C. On the next day, one-hundredth of the overnight cultures was spread onto solid YPD plates and incubated until colonies formed, and the number of colonies that carried the mouse mtDNA genomes was counted. One- hundredth of the remaining overnight cultures was taken again, reinoculated into fresh YPD medium and cultured overnight. This serial dilution and cultivation was repeated for 4 consecutive days, and the presence of mouse mtDNA genomes was assayed by PCR using mouse mtDNA-specific primers at each time point (days 1, 2, 3 and 4) [5, 12]. The average percentages of yeast cells containing the mouse mtDNA genomes are shown in Fig. 4. In the wild-type ABF2 donor and recipient strains (Fig. 4, black and white bars, respectively), more than 75% of the cells carried the mouse mtDNA genomes, which was a considerably high percentage of the yeast population and indicated that the mouse mtDNA was stably maintained in these yeast mitochondrial environments [5]. In the Tfam strain (Fig. 4, gray bars), however, the percentage of the yeast population containing the mouse mtDNA genomes decreased to between 15 and 25%, which was relatively low compared to the wild-type yeast strains. Although analyses of individual colonies isolated from the Tfam and wild-type ABF2 strains during this cultivation process showed that some cells lost the mouse mtDNA genomes and that the Tfam strain maintained the genomes at a lower rate than the wild-type strain, the proportion of cells carrying the mouse mtDNA in their mitochondria was relatively stable in these strains for many generations under the experimental conditions (Fig. 4).

## Discussion

In a previous report, a mouse Tfam-expressing yeast strain (Tfam strain) was confirmed to stably maintain full yeast mtDNA and thus survive on medium that required the cells to have active mitochondrial function [10]. These previous results indicated that the mouse Tfam protein can functionally replace the yeast mitochondrial nucleoid protein Abf2p and support mtDNA maintenance and mitochondrial biogenesis in yeast. In this report, I tested whether a yeast strain expressing the mouse Tfam protein can also stably maintain the entire mouse mtDNA genome, similar to maintaining the whole yeast mtDNA, in mitochondria. To transfer the mitochondria containing full mouse mtDNA genomes to the Tfam strain, a method for spheroplast fusion without the use of either nuclear or mitochondrial genetic markers of the partners was employed [13]. This method is based on the chemical inactivation of the cytoplasmic part of one parent cell by a chemical that has no effect on the genetic material. Enzyme activities were inhibited irreversibly by the chemical and were replaced by functional activities via fusion with a non-inactivated partner. Of the chemicals reported to inactivate only the cytoplasmic parts of cells, EMI (50 μg/ml) was shown to yield 100% inactivation, even at a high concentration (10^6^ cells/ml) of spheroplasts [13]. This method allows the transfer of cytoplasm and mtDNA from one yeast strain to another even though they have the same mating type and only one selectable difference. Using this fusion method, both haploid and diploid cells could be obtained because the donor cells (MCC109 ρ^-^) contained the *kar1-1* mutation, which permits efficient mitochondrial fusion but decreases nuclear fusion significantly (Fig. 3A) [17]. Thus, diploid yeast cells were removed from the fused cells by selection on uracil- and tryptophan-deficient selection media. Diploid cells can survive on both uracil- and tryptophan-deficient selection media, but haploid cells can grow only on uracil-deficient selection media (Fig. 3A). Using this strategy, the haploid Tfam strain containing the mouse mtDNA genome in its mitochondria was successfully isolated.

Due to incompatibility between the nuclear and mitochondrial genomes, the xenomitochondrial yeast strains that contained the entire mouse mtDNA genomes in their mitochondria did not have mitochondrial activity [5]. The mouse mitochondrial genome was maintained and replicated in a stable manner, for which the cells had to be grown under conditions that did not require mitochondrial activity, for example, on a fermentable carbon source [8]. For this reason, the mitochondrial protein content could be manipulated to a large degree without compromising cell viability. Thus, the flexibility of this system was exploited by adding nuclear-encoded mouse mitochondrial protein to these xenomitochondrial yeasts. In mammals, the mitochondrial genome exists in mitochondria as a protein/DNA complex, which is known as a nucleoid, and a major component of the nucleoid complex is mitochondrial transcription factor A (Tfam). Published data suggest that Tfam, which has relatively relaxed sequence specificity, wraps and packages the entire mitochondrial genome into the nucleoid structure [20, 21]. In addition to the DNA organizing activity of Tfam, which is analogous to the presumed function of its yeast homolog Abf2p, Tfam has been found to be essential for initiating mitochondrial transcription [22]. For these reasons, it was assumed that packaging the mouse mtDNA in yeast mitochondria with Tfam rather than Abf2p may greatly enhance the structural stability and biological activity of this DNA. Therefore, the Tfam strain in which the mouse *Tfam* gene was recombined into the yeast *ABF2* locus was confirmed to support mouse mtDNA maintenance activity in yeast mitochondria, although the activity was lower than that of the yeast homolog Abf2p (Fig. 4).

The finding presented here is believed to be important because it indicates that one could engineer a yeast genome to express a certain mammalian mitochondrial protein targeted to mitochondria and use this engineered yeast as a recipient of the xenomitochondria containing mammalian mtDNA genomes to analyze the interaction between mitochondrial proteins and the mtDNA and recapitulate various features of mammalian mtDNA replication and gene expression in mitochondrial environments [5]. In addition, it was surprising that the entire mouse mtDNA genomes were steadily maintained in the Tfam strain (Fig. 3), whereas no colonies that retained the mouse mtDNA genomes were isolated for the abf2Δ strain. These results are possibly due to the absence of the mtDNA organization activity of nucleoid proteins (Tfam or Abf2p). The yeast mtDNA maintenance activity was also rapidly lost when the Tfam ρ^+^ strain was subjected to growth on nonselective complete glucose medium in which the selection pressure to maintain mitochondrial function was absent (Figs. 2 and S1). Moreover, although the yeast mtDNA genomes were maintained in the abf2Δ strain when mitochondrial selection pressure was applied, the genomes were quickly lost when the selection pressure was abolished [10]. The results obtained here and in previous studies indicated that the mtDNA organization activity of nucleoid proteins was critically important for the maintenance and replication of mtDNA genomes in mitochondria and that the mouse Tfam protein can compatibly support the maintenance and replication of mtDNA genomes originating from mouse and yeast, respectively, in yeast cells.

The percentages of the yeast population retaining the mouse mtDNA genomes in the Tfam strain (15~25%) were lower than those in the wild-type yeast strains. Presumably, this may be because of the minimal activity of the mouse Tfam protein expressed in yeast [23]. When the Tfam protein is expressed in yeast, precise posttranslational regulation, such as acetylation and phosphorylation, would be limited, and thus, the functional activity of Tfam might be decreased due to improper modification of the protein [23]. This limited activity of Tfam might cause a smaller population of the Tfam-expressing strain to retain the mouse mtDNA genomes in yeast mitochondria. In addition, the limited Tfam activity may also cause rapid loss of yeast mtDNA genomes when the cells are placed in conditions that do not require active mitochondrial function (Fig. S1). The mechanism of mtDNA maintenance and replication in the Tfam strain is not exactly known; however, it is important to point out that the mtDNA organization activity of the mouse Tfam protein can support the maintenance of the entire mouse mtDNA as well as the yeast mtDNA in yeast mitochondria and that reasonable modification of these xenomitochondria with mouse proteins may enable us to analyze the interactions between mtDNA and mitochondrial proteins in essentially any desired manner as the technology is refined.

## Supplemental Materials



Supplementary data for this paper are available on-line only at http://jmb.or.kr.
