# Supplementary material for: Transfer of Xenomitochondria Containing the Entire Mouse Mitochondrial Genome into a Genetically Modified Yeast Expressing Mitochondrial Transcription Factor A
Source: J Microbiol Biotechnol. 2020 Jul 5;30(9):1290–6. doi: 10.4014/jmb.2004.04033 (PMC9728338; doi:10.4014/jmb.2004.04033)
Supplement: Supplementary file 1 [file JMB-30-9-1290-supple.pdf]

J. Microbiol. Biotechnol.
https://doi.org/10.4014/jmb.2004.04033

Supplementary Information

Transfer of xenomitochondria containing the entire mouse mitochondrial genome into a genetically modified yeast expressing mitochondrial transcription factor A

Young Geol Yoon *

Department of Biomedical Science, Jungwon University, 85 Munmu-ro, Goesan-eup, Goesan-gun, Chungbuk 28024, Republic of Korea

Supplementary Figure S1. Growth phenotypes of the wild-type and Tfam yeast strains. (A and B) Growth of the MCC109 ρ^+ wild-type and MCC109 Tfam ρ^+ strains on synthetic minimal medium containing ethanol and glycerol (SEG). The colonies that grew on the SEG medium possessed active mitochondrial function, and the red color shown in each colony was due to the *ade2-101* mutation. (C and D) Growth of the MCC109 ρ^+ wild-type and MCC109 Tfam ρ^+ strains on complete glucose medium (YPD). The MCC109 ρ^+ wild-type cells grown on the YPD medium showed a red color and maintained active mitochondrial function by retaining the yeast mtDNA (C). The majority of MCC109 Tfam ρ^+ cells grown on YPD medium turned white and lost mitochondrial function by losing the yeast mtDNA (D).

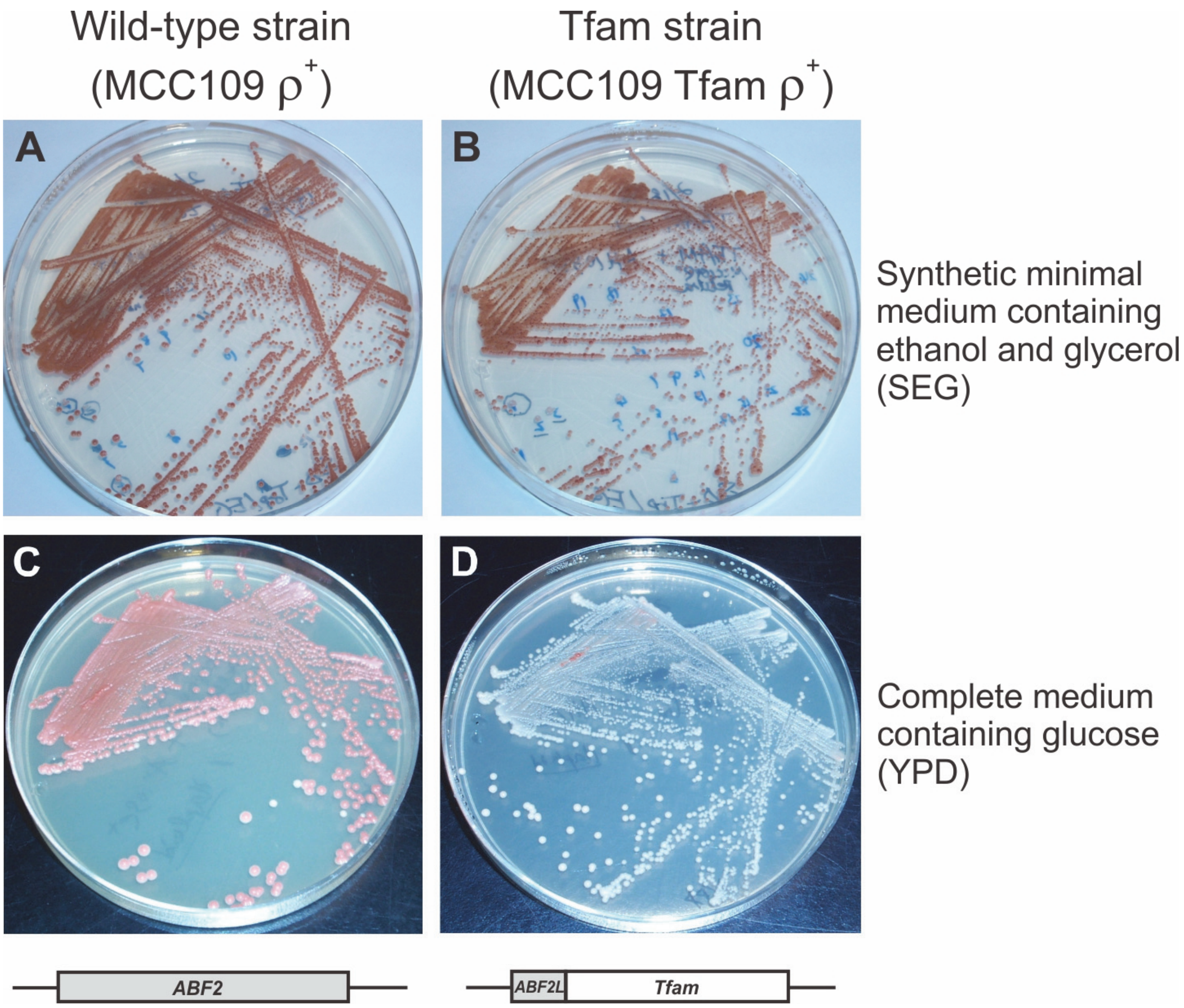
Wild-type strain
(MCC109 ρ+)
Tfam strain
(MCC109 Tfam ρ+)
A
B
Synthetic minimal medium containing ethanol and glycerol (SEG)
C
D
Complete medium containing glucose (YPD)
ABF2
ABF2L
Tfam
